# Diversity of forest structures important for biodiversity is determined by the combined effects of productivity, stand age, and management

**DOI:** 10.1007/s13280-023-01971-9

**Published:** 2024-01-02

**Authors:** Aino Hämäläinen, Kadri Runnel, Thomas Ranius, Joachim Strengbom

**Affiliations:** 1 Department of Ecology, Swedish University of Agricultural Sciences, Box 7044, 75007 Uppsala, Sweden; 2 Institute of Ecology and Earth Sciences, University of Tartu, J. Liivi 2, 50409 Tartu, Estonia

**Keywords:** Dead wood, Ecosystem quality, Even-aged forest management, Habitat trees, National Forest Inventory

## Abstract

In forests, the amount and diversity of structural features with high value for biodiversity, such as large trees and dead wood, are affected by productivity, stand age, and forest management. For efficient conservation of forest biodiversity, it is essential to understand the combined effects of these drivers. We used data from the Swedish National Forest Inventory to study the combined effects of productivity, stand age, and management for wood production on structures with high value for biodiversity: tree species richness, large living trees, dead wood volume, and specific dead wood types. Forest management changed the relationship between productivity and amount or diversity of some of the structures. Most structures increased with productivity and stand age, but decreased due to management. The negative effect of management was greatest for structures occurring mainly in high-productivity forests, such as deciduous dead wood. Thus, biodiversity conservation should target high-productivity forests to preserve these structures.

**Supplementary Information:**

The online version contains supplementary material available at 10.1007/s13280-023-01971-9.

## Introduction

Global conservation concern has led to widely accepted political targets addressing the allocation of land set aside for biodiversity conservation (e.g. CBD 2022). However, the current distribution of set-aside land is not always optimal for biodiversity conservation. Areas set aside for conservation are often located on low-productivity land (Gaston et al. 2008), whereas human land use, such as intensive agriculture or wood production that often has negative impacts on biodiversity, typically targets high-productivity land (e.g. Martin et al. 2020). This can be a risk for biodiversity conservation, since productivity and biodiversity are often correlated. The strength and shape of this correlation can vary depending on the scale of observation (Chase and Leibold 2002), or specific environmental and climatic conditions or disturbances (Grace et al. 2016). However, at large, biodiversity is often positively correlated with productivity (e.g. Chase and Leibold 2002; Gillman and Wright 2006; Cusens et al. 2012). Therefore, when mainly low-productivity land is set aside, biodiversity may be lost even if large areas are protected.

Here, we study the linkages between productivity, management, and biodiversity in Swedish forests. Productivity is here defined as the potential wood biomass production per unit area over a given time, measured in m^3^ ha^−1^ year^−1^ (Bontemps and Bouriaud 2014). The Swedish forests fall mainly within the boreal vegetation zone, with the exception of a smaller area of temperate forest in the southern parts of the country (Ahti et al. 1968). The boreal forests host relatively low tree species diversity: throughout the country, Norway spruce (*Picea abies* (L.) H. Karst.; hereafter spruce) and Scots pine (*Pinus sylvestris* L.; hereafter pine) are the dominant tree species in both protected and managed forests (Esseen et al. 1997; Hämäläinen et al. 2023). In these forests, the most common broadleaved trees are birches *Betula pendula* Roth. and *B. pubescens* Ehrh., and trembling aspen *Populus tremula* L., which in natural conditions occur mainly in younger successional stages or as an admixture with conifers in older forests (Gustafsson and Ahlén 1996; Hämäläinen et al. 2023). The temperate forests host a higher number of broadleaved tree species, including for example beech *Fagus sylvatica* L., ash *Fraxinus excelsior* L., oak *Quercus robur* L., and elm *Ulmus glabra* Huds. (Esseen et al. 1997).

A majority of the forest land in Sweden is managed for wood production. The prevailing management approach involves clear-cutting, which is typically succeeded by reforestation by planting and multiple thinning operations before the next round of clear-cutting (Bernes 2011). The average rotation period between two clear-cuttings is 60–120 years (Fries et al. 2015). Such management typically results in even-aged, structurally simplified forest stands consisting of one or two economically important tree species, which has significant negative effects on forest biodiversity (e.g. Esseen et al. 1997; Gauthier et al. 2015). The magnitude of these effects depends on the overall harvest level and, to lesser extent, on the choice of management strategies, such as rotation length or thinning intensity (Mäkelä et al. 2023). The negative effects on biodiversity are mitigated by protecting forests from management for wood production, and by applying conservation measures in managed forests, most commonly in the form of retention forestry where living trees and dead wood are retained at harvested sites (e.g. Gustafsson et al. 2012). As is the case with protected areas in general, forests selected for protection are often of low productivity (e.g. Fridman 2000), which can affect their conservation value (Hämäläinen et al. 2018). In contrast, conservation measures in managed forests do not generally consider productivity.

High structural diversity is considered crucial for maintaining high forest biodiversity, as it increases the availability of different habitat niches for forest-dwelling species (Chase and Leibold 2003). In boreal and temperate forests, especially important structural features are different types of dead wood and large living trees (Lindenmayer and Franklin 2002; Hekkala et al. 2023). An estimated 20–25% of all forest-dwelling species in Fennoscandia are dependent on dead wood (Siitonen 2001). A large proportion of all dead wood dependent species are insects and fungi, but there are also many bryophytes and lichens, and certain birds and mammals (e.g. Siitonen 2001). Thus, a higher amount and diversity of dead wood is typically connected with a higher diversity of many forest-dwelling taxa (e.g. Lassauce et al. 2011; Seibold et al. 2015). Large old trees provide important microhabitats, such as specific bark structures or cavities, which are essential for, e.g. epiphytic lichens and bryophytes, insects, and cavity-dwelling birds (e.g. Paillet et al. 2017; Kozák et al. 2023). Thus, the amount and diversity of such structures can function as a proxy for forest biodiversity (Larrieu et al. 2018; Hekkala et al. 2023). Therefore, biodiversity conservation measures often attempt to preserve or create these structures (Larrieu et al. 2018; Asbeck et al. 2021). For practical implementations, it is crucial to understand how productivity and forest management for wood production influence the quantity and diversity of these structures, and whether management alters the relationship with productivity.

Since forest productivity is here defined as the potential wood biomass production per unit area over a given time, higher productivity means a higher rate of wood formation, and as long as tree mortality is not affected by productivity, the total volume of living trees increases with productivity (e.g. Nilsson et al. 2002). In addition, high-productivity natural forests typically harbour a higher number of tree species (Mittelbach et al. 2001) with a wider variation in diameter than less productive forests (Liira et al. 2007). Thus, the structural diversity of living trees can be expected to increase with productivity. The amount of dead wood in a forest is dependent on both tree mortality and wood decay rates (Harmon et al. 1986). For forests where tree mortality and production are balanced, the volume of dead wood is positively correlated with productivity (Sippola et al. 1998; Nilsson et al. 2002; Ranius et al. 2004; Liira et al. 2007). However, the decay rate of dead wood can be lower in low-productivity forest, which may partly compensate for the slower formation of dead wood structures and thus increase dead wood volumes in such forests (Siitonen 2001).

Forest management for wood production, including the type of stand-replacing management that is practised in Sweden and other countries of Fennoscandia, is known to reduce the amount and diversity of many structures important for biodiversity (Siitonen 2001; Edenius et al. 2011; Lindenmayer et al. 2012). It is, however, unknown whether its effect differs depending on productivity. Nevertheless, since many management practices, such as the choice of tree species, frequency of thinning, and rotation periods, are often adjusted to productivity, interactive effects between forest management and productivity on the amount and diversity of structures seem likely. Another important factor known to affect the structures in forests is stand age (i.e. successional stage). The amount and diversity of structures changes with succession, both in forests regenerating after natural stand-replacing disturbances and after harvest (Siitonen 2001; Ranius and Kindvall 2004). However, the rate of change can depend on productivity. For example, higher growth rates in high-productivity forests can accelerate the development towards late-successional structures (Boucher et al. 2006; Larson et al. 2008). Similarly, tree species diversity typically changes with forest succession, but the exact pattern of change depends on productivity (Denslow 1980). Since forest management commonly alters tree species composition, management may also change the relationship between tree species diversity and productivity.

The aim of this study is to examine the combined effects of productivity, stand age, and forest management on the amount and diversity of structures important for forest biodiversity. The study is based on field data from Sweden, but the results are also applicable elsewhere in the boreal and temperate regions where even-aged forest management is practised. We use data from the Swedish National Forest inventory to analyse the amount and diversity of 13 different structural features, related to either living or dead trees. Specifically, we address whether forest management changes how the amount and diversity of these structures are related to productivity and stand age.

## Materials and methods

### Study area and data

We used data from the Swedish National Forest Inventory (NFI). The NFI is a repeated survey that includes both permanent and temporary study plots throughout Sweden. Thus, the data include all forested vegetation zones in Sweden, i.e. the temperate, hemiboreal, and boreal zones (Ahti et al. 1968). In this study, we used data collected in 2013–2017 from the permanent NFI plots. The permanent plots are circles with a radius of 10 m (area 314.16 m^2^), which are re-surveyed every fifth year. The plots are grouped in clusters that vary in size and number of plots. We included plots in productive forests (potential wood production > 1 m^3^ ha^−1^ year^−1^) with 100% forest land. We divided the plots into managed forests (i.e. forests managed for wood production) and protected forests. The latter included both formally protected forests and woodland key habitats (forests that are not formally protected, but have a high conservation value and are typically left unmanaged due to forest certification requirements). To keep the division between these categories clear, we excluded plots that according to the NFI survey were situated in managed forests but were classified as “natural forests” (i.e. no obvious traces of management), as well as plots within protected forests where signs of recent management operations were observed. Since there were very few plots < 60 years in protected forests, we excluded all plots where stand age was under 60 years. The final dataset included 4924 study plots, 4383 in managed and 541 in protected forests (Fig. S1).

### Studied structural features

We analysed the amount and diversity of different living tree and dead wood structures, which serve as important habitats for forest biota. A list of the studied structures is given in Table 1. The structures related to living trees were tree species richness, measured as the number of tree species per study plot, and the amount of large living trees (pine, spruce, or deciduous), measured as the number of trees per study plot. The studied dead wood structures were dead wood volume (m^3^ ha^−1^) and amount of different types of dead wood items (standing and downed coniferous and deciduous dead wood, measured as the number of dead wood items per study plot). Dead wood volume was positively correlated with the diversity of dead wood, estimated as the number of different types of dead wood objects per plot (standing/downed; deciduous/conifer; hard/soft dead wood in different diameter classes; Spearmans correlation *r* = 0.71). Thus, dead wood volume reflects both the amount and diversity of dead wood.

**Table 1:** The studied structural features, based on Lundström et al. (2011)

Variable	Inclusion criteria
*N* of tree species per plot	> 10 cm DBH living trees
*N of large living trees per plot*	
Norway spruce	> 40 cm DBH
Scots pine	> 40 cm DBH
Deciduous trees	> 40 cm DBH
DW volume (m^3^ ha^−1^)	> 10 cm diameter, min. 1 m long downed DW
*N of DW items per plot*	
Downed conifer DW	10–20 cm or > 20 cm diameter, min. 1 m long
Standing conifer DW	10–20 cm or > 20 cm DBH, with or without broken top
Downed deciduous DW	10–20 cm or > 20 cm diameter, min 1 m long
Standing deciduous DW	10–20 cm or > 20 cm DBH, with or without broken top

*DW* dead wood, *DBH* diameter at breast height

Note that small (10–20 cm) and large (> 20 cm) diameter DW items were studied separately

### Explanatory variables

The structural features were modelled as a function of management (managed or protected), forest productivity, and stand age. Forest productivity was obtained directly from the NFI data and refers to forest site productivity, i.e. the potential for tree growth (measured as m^3^ ha^−1^ year^−1^; Skovsgaard and Vanclay 2008). In the NFI, productivity is estimated using one of two methods. In homogenous > 30 years old stands, a “dendrometric” approach (Skovsgaard and Vanclay 2008) is used: the top height of the dominant tree species at a given stand age is translated to productivity in m^3^ ha^−1^ year^−1^ (Hägglund and Lundmark 1977). In other types of stands, productivity is calculated based on soil and humidity characteristics, and ground vegetation (Hägglund and Lundmark 1977). Both methods give equivalent productivity measures that are independent of stand age, and account for differences in growth conditions along the latitudinal and altitudinal gradient. In addition, both measures are comparable for stands dominated by different tree species, because the equations take into account tree species-specific growth rates. The range of productivity rates at the study plots was 1–16 m^3^ ha^−1^ year^−1^. Stand age was obtained from the NFI data and calculated as the mean tree age weighted by basal area.

### Analyses

All statistical analyses were conducted in R (R Core Team 2019). We modelled the structural features with generalized linear mixed models (GLMMs) using the lme4 package (Bates et al. 2015). Poisson distribution (with a log link) was used to model tree species richness, and negative binomial distribution (log link) to model the amount of large living trees and dead wood items. Both are suitable for modelling count data. The volume of dead wood was modelled using a zero-inflated GLMM with gamma distribution (log link) from the glmmTMB package (Brooks et al. 2017). This type of model is suitable for analysing continuous data with large numbers of zeros (in this case: plots with no dead wood). In all models, we included management (binary variable: managed or protected), productivity, and stand age (continuous variables) as predictors. Squared terms for productivity and stand age were included to account for possible nonlinear relationships. In addition, we included all possible two-way interactions: productivity × management, productivity × age, and age × management. The squared and interaction terms were kept in the final models only if this resulted in a decrease in AIC value > 2 (Burnham and Anderson 2004). To account for possible spatial autocorrelation among plots located within the same cluster, we included the clusters as random variables in all models.

We standardized all continuous predictor variables prior to analysis, and assessed adequate residual distribution using the DHARMa package (Hartig 2022). Model results were plotted using packages coefplot2 (Lander 2022), ggeffects (Lüdecke 2018), and ggplot2 (Wickham 2016). We plotted the relationships separately for managed and protected forests, and for two stand ages (60 and 120 years, hereafter “younger” and “older” stands). These two fixed ages were chosen to illustrate the values at the lower and upper parts of the interval of normal cutting ages in Sweden (Fries et al. 2015, see above).

## Results

**Fig. 1:**
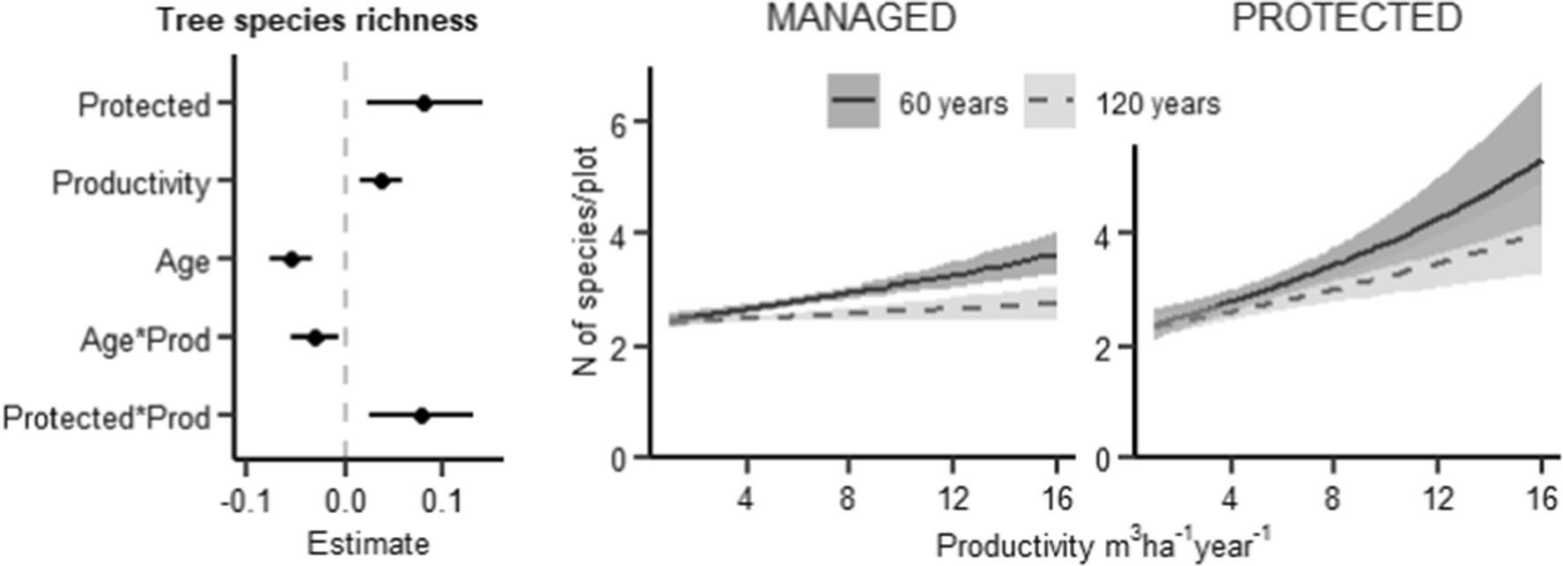
Results of a GLMM modelling the species richness of living trees (*N* of tree species per study plot). Left: Standardized coefficient estimates and 95% CIs (horizontal lines). Managed forests are used as a reference category for management. Middle and right: Regression lines from the model, depicting mean relationships and 95% CIs (shaded areas) between tree species richness and productivity in managed and protected forests, respectively. Regression lines are conditional of holding stand age at 60 years (unbroken line) and 120 years (dashed line); other explanatory variables are held constant at the median level. The lines are plotted on the scale of the original response

**Fig. 2:**
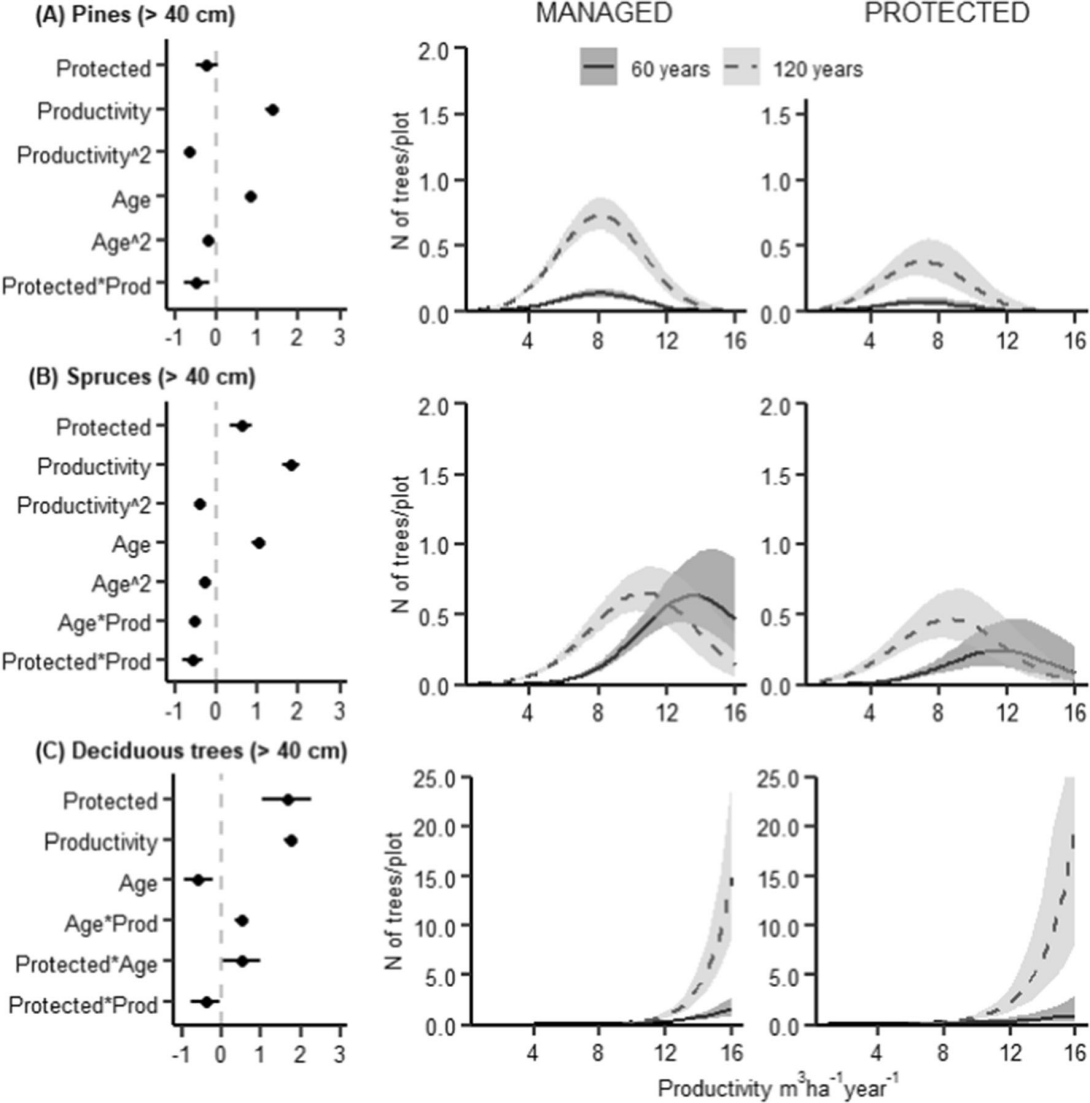
Results of a GLMM modelling the amount of large living trees (DBH > 40 cm) at the study plots (measured as N of trees per plot). Modelled separately for **A** Scots pine, **B** Norway spruce, and **C** deciduous trees. For further explanations, see Fig. 1

We found a significant interaction between management and productivity for six out of the thirteen studied structural features, i.e. management changed the relationship between productivity and the amount or diversity of these structures. Tree species richness, the amount of large deciduous trees, and the amount of small deciduous standing dead wood increased with productivity, but this increase was notably smaller in managed forests (Figs. 1, 2C, and 5A). Moreover, the amount of large spruce and pine trees and small coniferous dead wood was highest at medium productivity levels, but in managed forests the peaks occurred at lower productivity levels than in protected forests (Figs. 2A, B and 4A). We did not observe significant interactions between management and productivity for the other seven structural features. A significant interaction between stand age and management was found for only one structure: the amount of large deciduous trees (a greater increase with age in protected than managed forests, Fig. 2C). Generally, managed forests had a lower amount or diversity of most studied structures than protected forests. An exception was the amount of large living pine and spruce trees, which were more common in managed than protected forests (Fig. 2A, B).

Most structural features increased with productivity, or peaked at medium productivity, and increased with stand age. There was often a significant effect of the interaction between productivity and stand age on the structural features. The species richness of living trees increased with productivity and decreased with stand age at higher productivity levels (Fig. 2). Large living pine and spruce trees (DBH > 40 cm) had a hump-shaped relationship with productivity (Fig. 2A, B), whereas large living deciduous trees increased with productivity and were nearly absent in forests with productivity < 8 m^3^ ha^−1^ year^−1^ (Fig. 2C). Large pine and deciduous trees increased with stand age, and for deciduous trees, the increase with age was larger at higher productivity levels (Fig. 2A and C). The effect of stand age on the amount of large spruce trees depended on both productivity and management: their amount increased with age at lower productivity levels, especially in protected forests, but decreased with age at higher productivity levels (Fig. 2B).

**Fig. 3:**
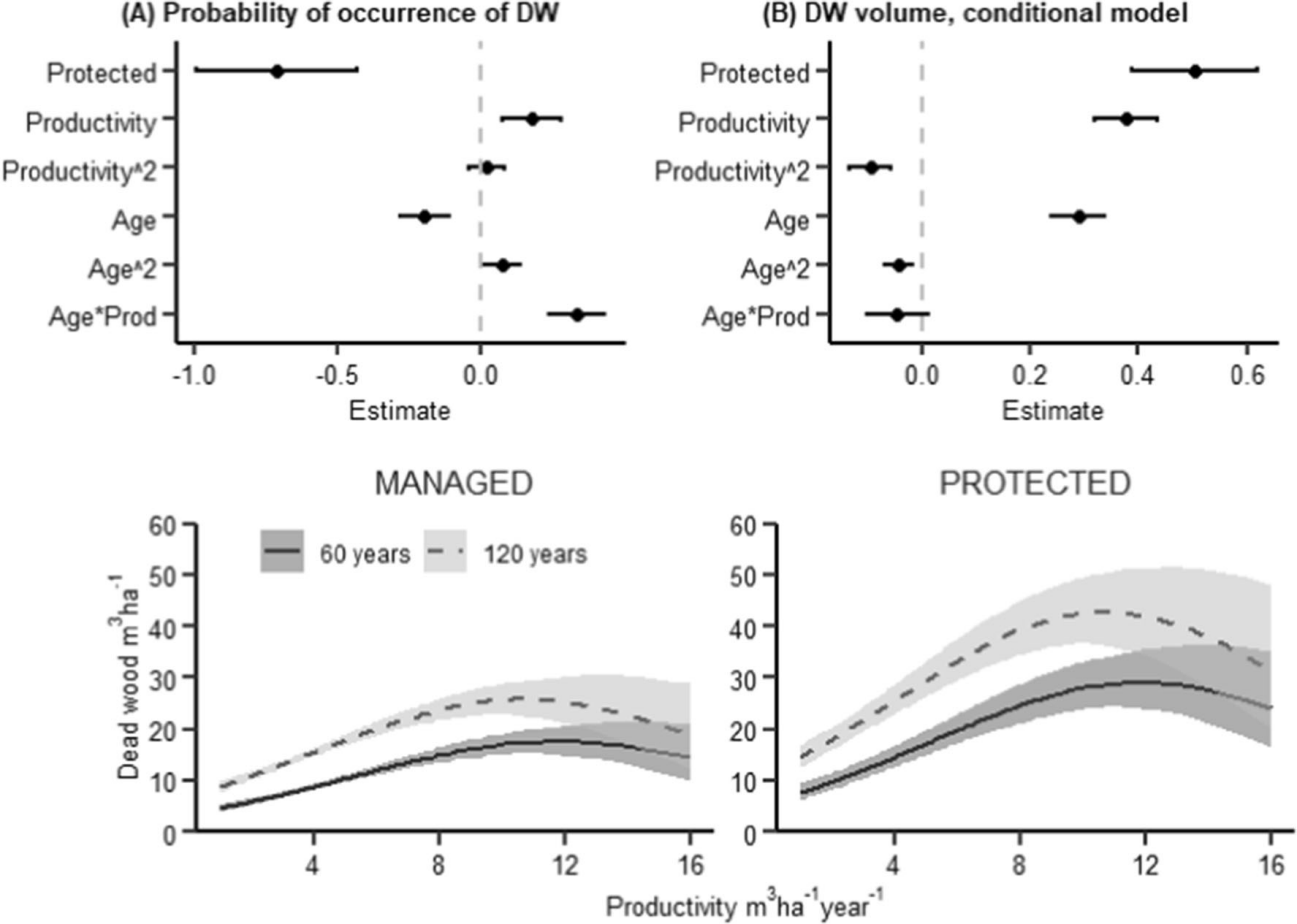
Results of a zero-inflated GLMM modelling dead wood volume (m^3^/ha). The zero-inflated model consists of two parts: a binomial model that predicts the probability of occurrence of dead wood, and a conditional model with gamma distribution that predicts the amount of dead wood when it is > 0. Top row: model coefficients with 95% CIs (horizontal lines) for **A** binomial model and **B** conditional model. Bottom row: Regression lines from the model, depicting mean relationships and 95% CIs (shaded areas) between dead wood volume and productivity in managed and protected forests, respectively. For further explanations, see Fig. 1

**Fig. 4:**
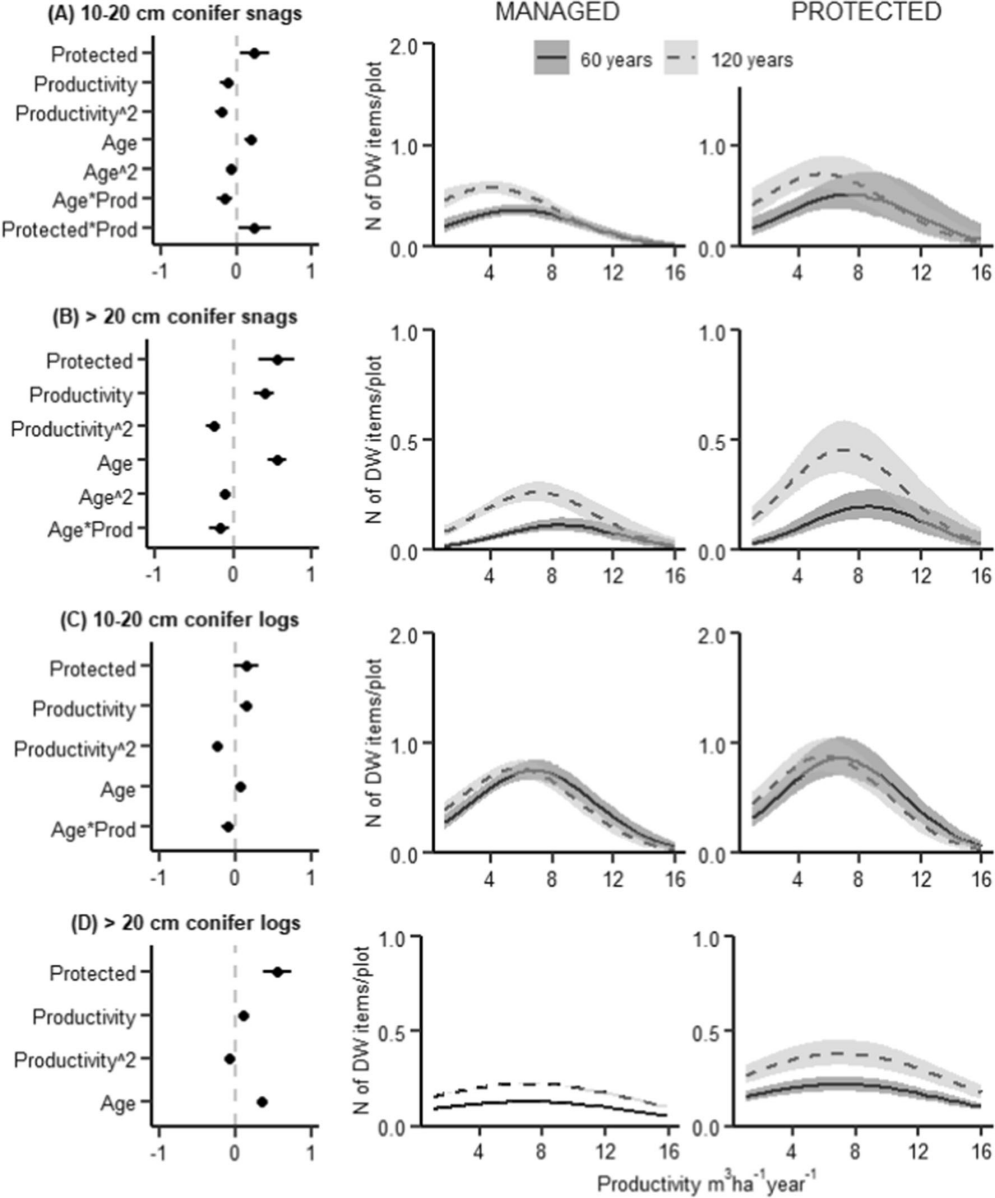
Results of a GLMM modelling the amount of coniferous dead wood at the study plots (as N of dead wood items per plot). Modelled separately for (A) standing dead wood with diameter 10–20 cm, (B) standing dead wood with diameter > 20 cm, (C) downed dead wood with diameter 10–20 cm, and (D) downed dead wood with diameter > 20 cm. For further explanations, see Fig. 1

**Fig. 5:**
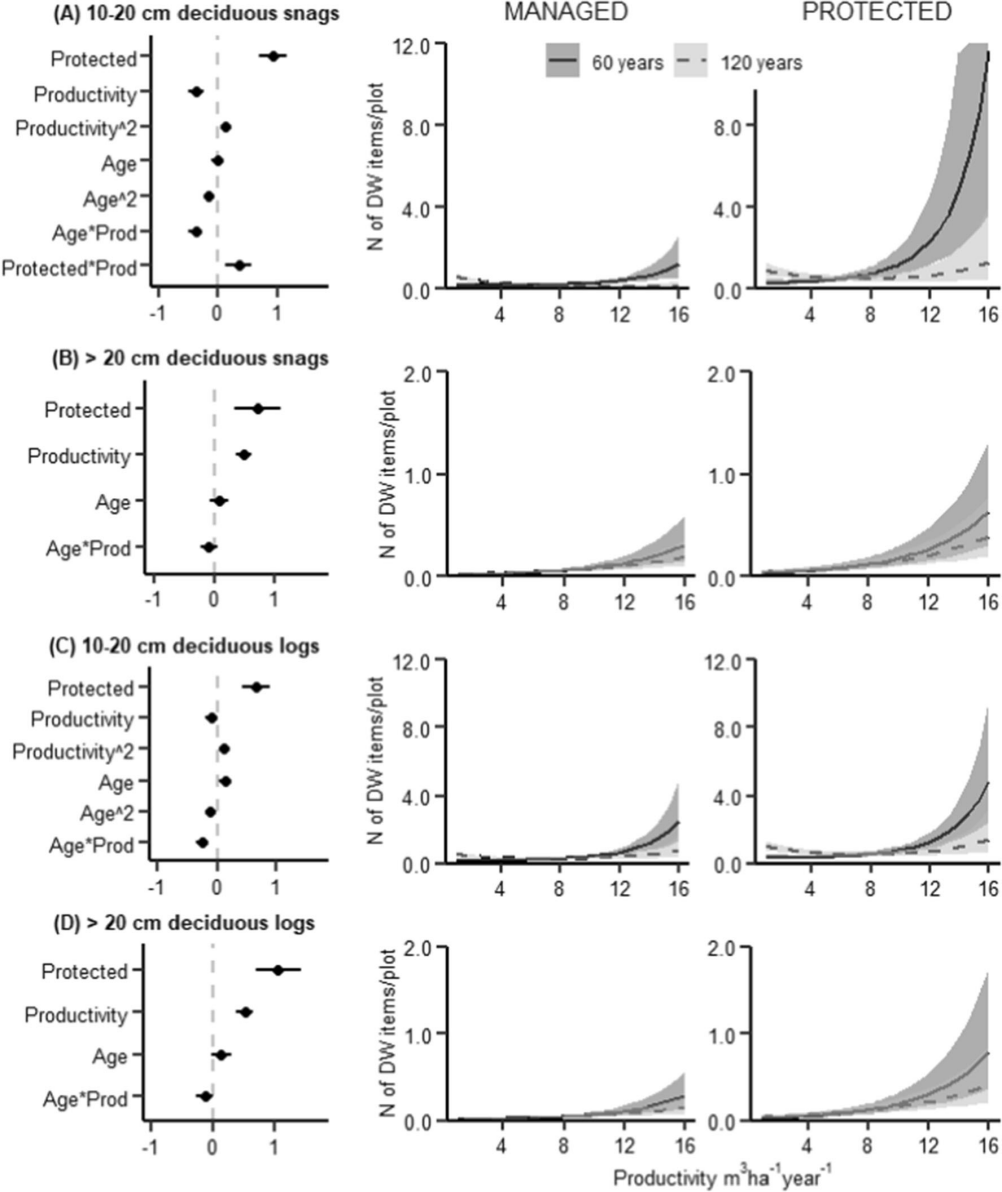
Results of a GLMM modelling the amount of deciduous dead wood at the study plots (as N of dead wood items per plot). Modelled separately for (A) standing dead wood with diameter 10–20 cm, (B) standing dead wood with diameter > 20 cm, (C) downed dead wood with diameter 10–20 cm, and (D) downed dead wood with diameter > 20 cm. For further explanations, see Fig. 1

The volume of dead wood increased with stand age and had a hump-shaped relationship with productivity, with the highest dead wood volumes found in forests with a productivity of 8–12 m^3^ ha^−1^ year^−1^ (Fig. 3). The amount of conifer dead wood was most abundant at medium productivities (Fig. 4), while deciduous dead wood increased with productivity and was rare at productivity levels < 8 m^3^ ha^−1^ year^−1^ (Fig. 5). The amount of conifer dead wood, especially standing dead wood, increased with stand age at low productivities. In contrary, deciduous dead wood was either unaffected or, in case of small standing dead wood, decreased with stand age.

Median productivity of the included study plots was 4.3 m^3^ ha^−1^ year^−1^ (quartiles 3.0, 6.5), with higher values in managed (median 4.4; quartiles 3.1, 6.8) than in protected forests (median 3.1; quartiles 2.2, 5.1). For more detailed descriptive statistics, see Table S1.

## Discussion

### Combined effects of management, productivity, and stand age

The amount and diversity of most studied structures were lower in managed forests, as expected (e.g. Siitonen 2001; Lindenmayer et al. 2012). We found that management altered the relationship between productivity and the amount or diversity of six out of the 13 studied structures. Management decreased the positive effect of productivity on tree species richness, the amount of large deciduous trees, and the amount of small-diameter standing deciduous dead wood. It also increased the amount of large spruce and pine trees in low- and medium-productivity forests, and decreased the amount of small-diameter standing conifer dead wood in medium and high productivity levels.

In protected forests, tree species richness increased with productivity (Fig. 1), concurring with previous studies (Paquette and Messier 2011; Liang et al. 2016). In contrast, in managed forests tree species richness was low overall, i.e. independent of productivity and on the same level as in low-productivity protected forests. Typically, forest management aims at creating stands of one or two commercially valuable tree species despite productivity (Puettmann et al. 2012), and thus decreases the positive effect that productivity has on tree species richness. Tree species richness was further affected by stand age: the positive effect of productivity was clear in younger stands, but not in the older ones. One possible explanation for this is that in high-productivity old forests, the competition from one or a few dominating tree species is higher than in younger forests with the same productivity.

In the case of large deciduous trees, management had a significant interaction with both productivity and stand age. The amount of large deciduous trees increased with productivity and stand age, as can be expected, but in both cases, the increase was greater in protected than managed forests. This result can be attributed to forest management that favours conifers over deciduous trees, both by planting conifers and by removing deciduous trees especially during clearing (Bernes 2011). In general, large deciduous trees and deciduous dead wood were the structures most strongly and negatively affected by management (Figs. 2C, 5). In addition to forest management favouring conifers, another factor that can contribute to this result is that in Sweden, forests dominated by deciduous trees are considered to have particularly high conservational values (Bernes 2011). Such forests are therefore likely to be prioritized for conservation, which can have led to a higher share of deciduous forests in protected than managed land and contributed to the higher amounts of deciduous tree structures in protected forest. Deciduous trees were also strongly affected by productivity: both large living deciduous trees and deciduous dead wood were almost absent in forests with low productivity, and increased notably in protected forests with productivity over 12 m^3^ ha^−1^ year^−1^. In Sweden, forests with such a high productivity occur mainly in the southernmost parts of the country, which belongs to the temperate vegetation zone, and hosts several broadleaved tree species that are absent from the boreal regions of the country (Bernes 2011).

Management changed the relationship between productivity and both coniferous and deciduous small-diameter standing dead wood (Figs. 4A and 5A). Coniferous dead wood of this type was most abundant at medium productivity levels, but management decreased its amount especially in more productive forests. In turn, deciduous dead wood increased with productivity, but the increase was notably smaller in managed than protected forests. These results can be attributed to a higher intensity of management, with a higher frequency of thinning and clear-cutting operations, in more productive stands. Generally, harvesting decreases standing dead wood more than downed (e.g. Graves et al. 2000; Rosenvald et al. 2018). Furthermore, forest management aims at decreasing the level of self-thinning, which is a process that increases especially the amount of standing dead wood. As a consequence, the proportion of standing dead wood is typically higher in protected than managed forest (e.g. Ekbom et al. 2006). Concurrently, the effects of management were more pronounced for standing than downed dead wood, both in the case of coniferous (Fig. 4) and deciduous (Fig. 5) dead wood.

Management influenced the effect of productivity on the amount of large living pine and spruce trees (Fig. 2A, B). These were the only two structures for which the effect of management was positive, which was expected as pine and spruce are the most important commercial tree species in Sweden, and management specifically aims to promote their growth. However, the increased amount of large conifers in managed forests is likely of limited importance for biodiversity, since the trees are typically harvested before they reach a sufficiently old age to develop specific microhabitats that host specialized species (Paillet et al. 2017; Kozák et al. 2023), and since many species that inhabit living trees require longer time for colonization than the current rotation periods allow (Kuusinen and Siitonen 1998; Marmor et al. 2011). The positive effect of management also decreased with productivity, thus changing the relationship between productivity and the amount of large conifers. This suggests that forest management for high wood production increases tree growth more in low- and medium-productivity forests than in high-productivity forests, which are likely to host more large trees even in the absence of management.

Finally, management appeared to alter the effect of stand age on the amount of large spruces, although the interaction between management and stand age was not significant (Fig. 2B). In protected forests, the amount of large spruce trees increased with stand age, but management tended to decrease this effect, suggesting that forest management increases the growth rates of spruces and allows them to reach a diameter of > 40 cm faster in managed than in protected forests.

### Effects of productivity

The effects of productivity on the amount and diversity of most structures were as expected, with one surprising exception, namely the total dead wood volume that showed a hump-shaped relationship with productivity (Fig. 3). This contrasts with previous studies (both empirical studies and simulations) that suggest that dead wood volume generally increases with productivity (Sippola et al. 1998; Ranius et al. 2004; Liira and Kohv 2010). The low dead wood volume in high-productivity forests can be a legacy effect from historical management. Most forests in Fennoscandia, including currently protected forests, are influenced by past management, such as selective logging (e.g. Ericsson et al. 2005; Kyaschenko et al. 2022). This is likely also the case for the protected forests in our study. Historical tree harvest also in protected forest can explain why the difference in dead wood volume between managed and protected forests was much smaller than what has been reported by previous studies (e.g. Siitonen 2001). Previous studies have also indicated that the effects of this past management are stronger in high-productivity forests (e.g. Storaunet et al. 2005), which can explain the lower dead wood volume in high-productivity forests.

### Implications for conservation

We found that forest management altered the relationship between some of the studied structures and productivity or stand age. Since the studied structural features could be seen as proxies for biodiversity, our results have important implications for biodiversity conservation in Sweden as well as elsewhere in the boreal and temperate regions where even-aged forest management is applied. Because a broader range of productivity levels is likely to host varying plant communities and provide a wider array of habitats, our results underline the need to protect forests across different productivity levels. Moreover, our results highlight the importance of protecting high-productivity forests from production forestry. We found that management had the strongest negative effects on structures that occurred predominantly in the most productive forests: large living deciduous trees and deciduous dead wood. Thus, we conclude that conservation efforts should be directed primarily towards high-productivity forests to maintain the structures and species typical to them. The imperative to prioritize high-productivity forests is emphasized by the recognition that these forests are inadequately represented within protected areas (Fridman 2000; Gaston et al. 2008). Consequently, there is a compelling need to prioritize high-productivity forests among new protected areas to counterbalance the extensive protection of low-productivity forests. In addition, our results suggest that protected high-productivity forests are commonly impacted by past management and therefore impoverished of certain structures, which lowers their conservation value. They may therefore require restoration management that promotes the development of the structural diversity lost due to past forestry.

Since the amount of protected areas is generally too small to alone maintain biodiversity, area conservation must be accompanied by conservation measures in production forests, especially in those of high productivity. Increased rotation lengths, i.e. increased intervals between clear-cut harvests, have been suggested as a measure to increase structural diversity and thereby promote biodiversity in managed forests (e.g. Roberge et al. 2018). However, based on our results, this might be an inefficient strategy, since the increase in the diversity and amount of most structures was rather small between 60- and 120-year-old managed stands. Therefore, it will likely be more effective to apply measures such as retention forestry that intentionally create and maintain important structures in managed forests, so that theses structures are present already in earlier forest successional stages. Moreover, to make the managed forests more similar to natural forests, the amount of deciduous trees and dead wood should be increased. This can be achieved by either increased focus on deciduous trees when practising green tree retention, or by applying silvicultural management practices that promote mixed species stands during planting, clearings, and thinning operations.

### Supplementary Information

Below is the link to the electronic supplementary material.

Supplementary file1 (PDF 504 KB)
