# Supplementary material for: Diversity of forest structures important for biodiversity is determined by the combined effects of productivity, stand age, and management
Source: Ambio. 2024 Jan 2;53(5):718–29. doi: 10.1007/s13280-023-01971-9 (PMC10992050; doi:10.1007/s13280-023-01971-9)
Supplement: Supplementary file 1 — Supplementary file1 (PDF 504 KB) [file 13280_2023_1971_MOESM1_ESM.pdf]

Ambio

Supplementary Information

This supplementary information has not been peer reviewed.

Title: **Diversity of forest structures important for biodiversity is determined by the combined effects of productivity, stand age, and management**

Aino Hämäläinen, Kadri Runnel, Thomas Ranius, Joachim Strengbom

Figure S1. The location of the study plots in Sweden and their productivities (classified in quantiles).

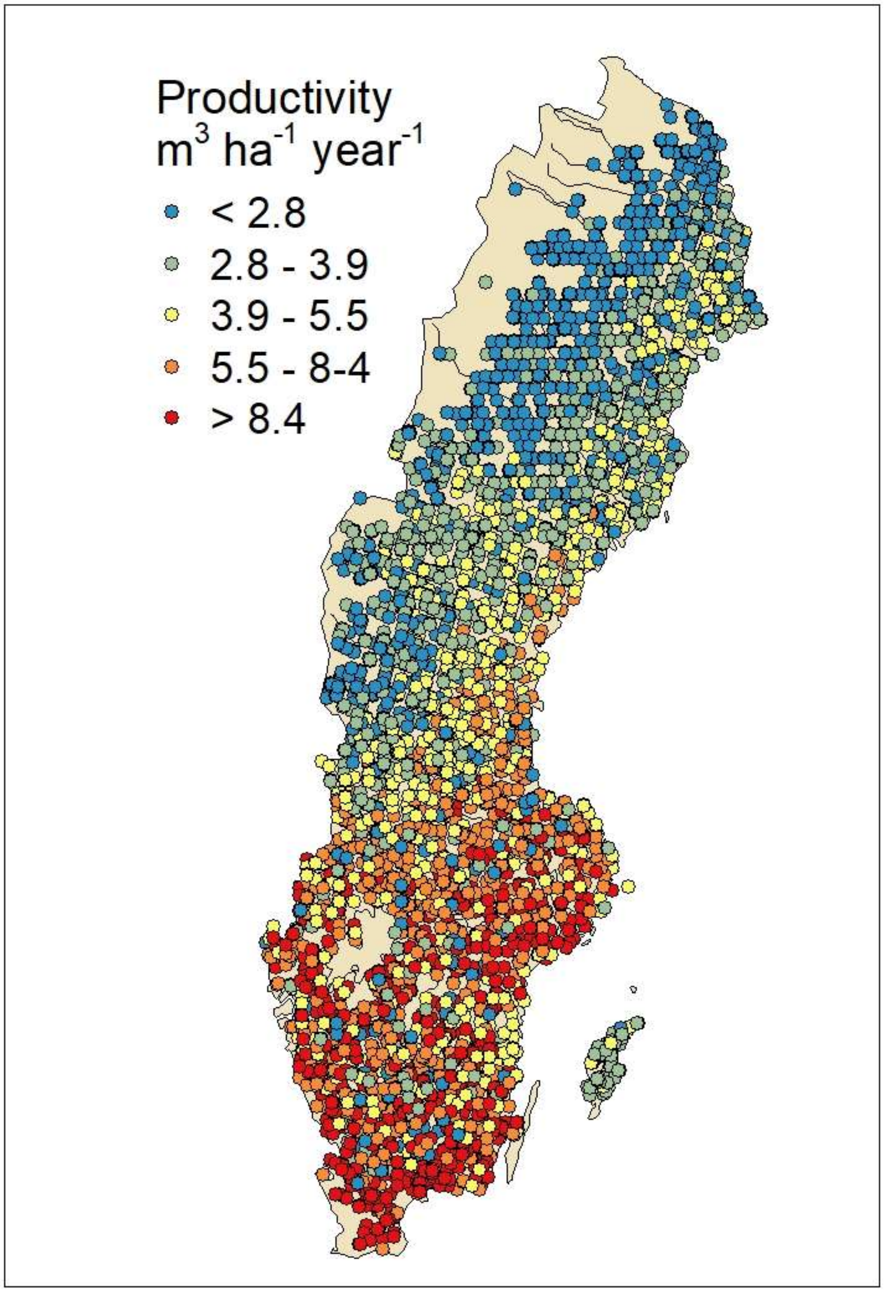

Table S1: Descriptive statistics of the study plots

	Managed forests		Protected forests	
**Explanatory variables**	Mean + SD	Range	Mean + SD	Range
Productivity	5.3 + 2.9	1 – 16	4.3 + 2.9	1.2 – 14.6
Stand age	102.1 + 33.1	60 – 280	142.9 + 42.6	60 – 285
Length of growing season	168.9 + 27.1	100 – 220	153.2 + 29.7	110 – 220
